# Definitive Airway Management in Sepsis-Related Airway Edema Following a Failed Intubation: An Anesthesia-Guided Approach to High-Risk Airway Management

**DOI:** 10.7759/cureus.105069

**Published:** 2026-03-11

**Authors:** Mahsum Jafri, Zuhayr Khan, Gurubasanagouda Payappagoudar, Hunnan Jafri, Constantino G Lambroussis

**Affiliations:** 1 Internal Medicine, Lake Erie College of Osteopathic Medicine, Elmira, USA; 2 General Medicine, Lake Erie College of Osteopathic Medicine, Elmira, USA; 3 Anesthesiology, Arnot Ogden Medical Center, Elmira, USA; 4 Biology, Adelphi University, Garden City, USA; 5 Osteopathic Medicine/Family Medicine, Lake Erie College of Osteopathic Medicine, Elmira, USA

**Keywords:** airway edema, airway management, anesthesiology, critical illness, difficult airway, failed intubation, fiberoptic bronchoscopy, sepsis, septic shock, video laryngoscopy

## Abstract

Severe infection and sepsis can significantly alter airway anatomy through inflammation, edema, and tissue friability, complicating definitive airway management in critically ill patients. These challenges are further magnified in individuals with prior failed intubation or airway trauma, where the margin for error is narrow, and loss of a functioning airway can be catastrophic. We report the case of a critically ill patient with influenza A-associated acute hypoxic respiratory failure and septic shock. Our patient required definitive airway management in the setting of severe airway edema and prior failed intubation complicated by cardiac arrest. The patient arrived with an existing tracheal airway and extensive airway inflammation, which limited standard airway visualization. Due to the high risk of airway loss, a staged, anesthesia-led approach was employed. The main goal was to preserve the existing airway while escalating visualization techniques. The initial video laryngoscopy was not successful in earlier attempts due to diffuse airway edema. Therefore, alternative video laryngoscopy improved visualization and allowed for controlled fiberoptic bronchoscopy, enabling accurate positioning of the tube and confirmation. This case demonstrates the importance of preserving an existing airway and using staged visualization techniques when managing complex airways with severe infection and airway inflammation.

## Introduction

Airway management in critically ill patients remains one of the highest-risk responsibilities of anesthesiologists, particularly in the setting of severe infection and sepsis. Systemic inflammation associated with sepsis can lead to airway edema, mucosal inflammation, and tissue friability, all of which may significantly distort upper airway anatomy and impair visualization [1]. These changes are often unpredictable and may persist despite clinical stabilization. They can also render standard airway assessments unreliable or unobtainable [1].

Patients with airway inflammation related to severe infection and sepsis are especially vulnerable to hypoxia during airway manipulation due to reduced physiologic reserve [2]. When this is compounded by prior airway trauma or failed intubation, the risk of airway loss increases substantially. In such cases, repeated or blind attempts at airway instrumentation can worsen edema, increase bleeding, and further narrow the margin for safe intervention [1]. As a result, anesthetic management must prioritize maintenance of oxygenation and ventilation while minimizing additional airway injury.

Although numerous airway devices and algorithms exist, no single technique is universally effective in the presence of severe airway edema and distorted anatomy. Successful airway management in these settings depends less on device selection and more on anesthesiologist judgment, anticipation of difficulty, and flexibility in technique [3]. A staged, visualization-first approach that preserves an existing airway while escalating methods in a controlled manner may offer a safer alternative to rapid airway exchange or repeated attempts with a single modality [3]. Current difficult airway guidelines, including those from the American Society of Anesthesiologists (ASA), emphasize oxygenation preservation, limitation of repeated attempts, and early use of advanced visualization techniques in anticipated difficult airways [4]. 

In this case report, we describe a patient with influenza A-associated acute hypoxic respiratory failure and septic shock. He required definitive airway management following prior failed intubation, complicated by cardiac arrest. This case illustrates how sepsis-related airway inflammation influences anesthetic decision-making and highlights the importance of deliberate sequencing, device adaptability, and airway preservation in the management of high-risk airways.

## Case presentation

A 54-year-old man presented to the emergency department with progressive shortness of breath. He was diagnosed with influenza A infection complicated by acute hypoxic respiratory failure and septic shock. Despite initial supportive management, his respiratory status deteriorated, and urgent airway intervention became necessary.

**Figure 1:**
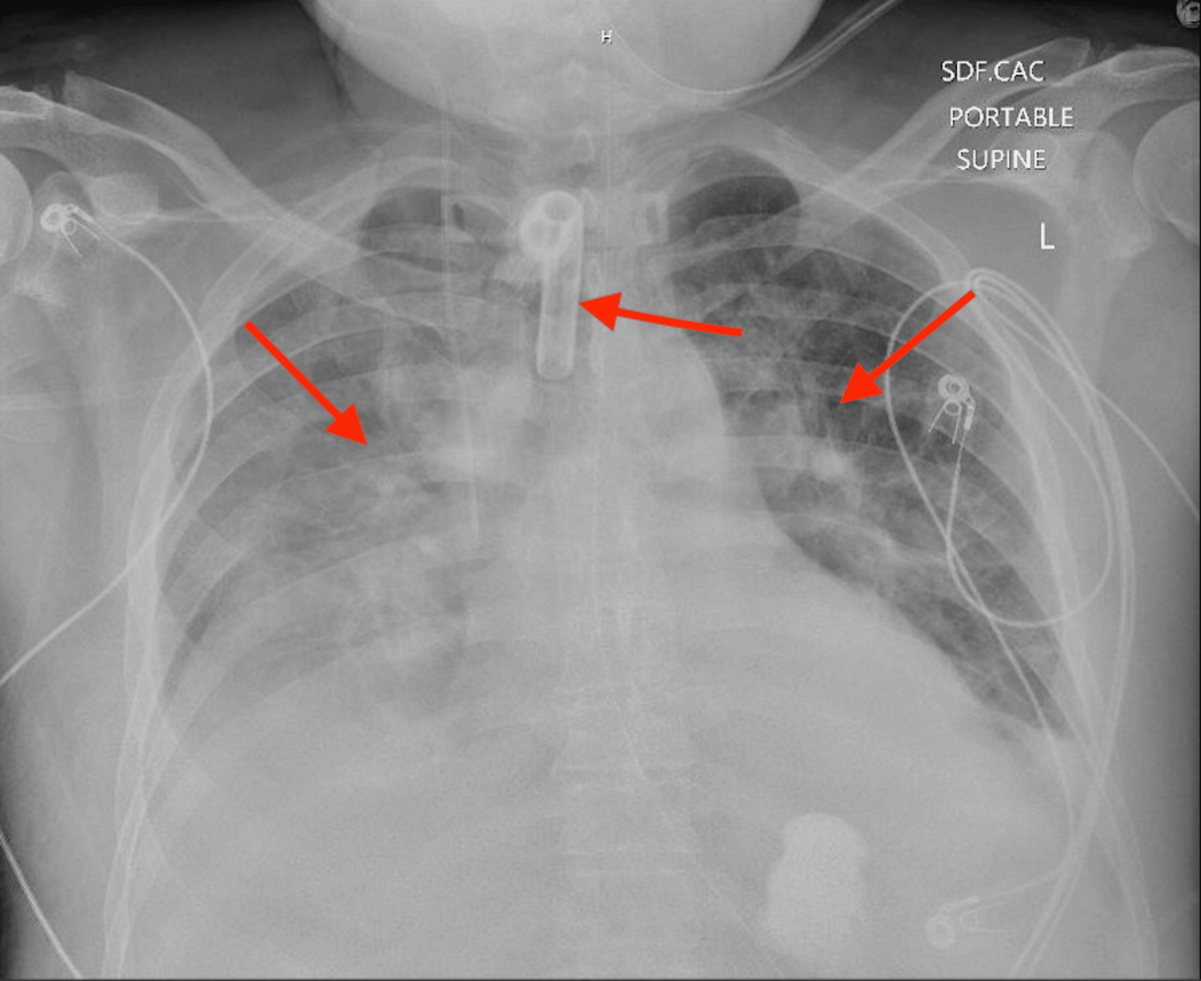
Portable chest radiograph demonstrating a cuffed tracheostomy tube (middle arrow) with bilateral diffuse pulmonary infiltrates (left and right arrows), consistent with acute hypoxic respiratory failure in the setting of influenza A and septic shock.

An attempt at oral endotracheal intubation was unsuccessful and resulted in worsening hypoxemia followed by a brief cardiac arrest. Succinylcholine was not administered during airway management, and neuromuscular blockade was achieved with rocuronium. The patient required cardiopulmonary resuscitation and administration of epinephrine, after which return of spontaneous circulation was achieved. An emergent cricothyrotomy was performed following cardiac arrest to secure the airway. The patient was subsequently taken to the operating room, where a formal 9.0-cuffed tracheostomy was placed and the cricothyrotomy tube was removed. The tracheostomy tube remained in place throughout his subsequent intensive care unit course (Figure 1).

Following stabilization, the patient was admitted to the intensive care unit, where he remained mechanically ventilated, sedated, and paralyzed. He was not neurologically intact and was unable to participate in an airway examination. His course was notable for significant airway edema and friable tissue, likely related to recent airway manipulation in the setting of severe infection and systemic inflammation. 

At the time of the current procedure, the patient arrived in the operating room with a cuffed tracheostomy tube in place, functioning as a stable airway. At the time of transfer, blood pressure was 112/72 mmHg, pulse 104 beats per minute, respiratory rate 16 breaths per minute, temperature 37°C, and oxygen saturation 93%. Given his prior failed intubation complicated by cardiac arrest and the presence of severe airway edema, the airway was anticipated to be extremely challenging. A formal airway assessment, including Mallampati classification and neck mobility evaluation, was not feasible due to sedation and mechanical ventilation.

For induction and airway management, the patient received midazolam 2 mg, fentanyl 50 mcg, lidocaine 50 mg, propofol 80 mg, and rocuronium 50 mg for neuromuscular blockade. Dexamethasone 4 mg was administered to mitigate airway edema. Hemodynamic support included phenylephrine infusion (200 mcg total administered from a 40 mcg/mL solution). Additional medications included cefazolin 2 g, ondansetron 4 mg, sugammadex 200 mg for reversal, and 300 mL of lactated Ringer's solution intraoperatively. 

Initial airway evaluation using a GlideScope video laryngoscope (Verathon Inc., Bothell, WA, USA) with a size 4 blade was unsuccessful due to diffuse airway edema, with swollen tissue obscuring normal anatomic landmarks. The blade curvature and limited maneuverability in the setting of severe soft tissue swelling made visualization difficult. A McGrath video laryngoscope (Medtronic, Minneapolis, MN, USA) with a size 4 blade was subsequently used, providing improved visualization likely due to its slimmer blade profile and improved maneuverability within the edematous airway. Although the airway remained markedly edematous, structures distal to the swelling were identifiable. Serosanguineous fluid and bloody secretions were noted and suctioned.

Given the distorted anatomy and high risk of airway loss, a fiberoptic bronchoscope with a size 6.0 endotracheal tube attached was used while maintaining visualization with the McGrath laryngoscope. The fiberoptic scope was advanced and visualized entering the airway, with improved airflow observed following deflation of the existing tracheostomy tube's cuff. The new endotracheal tube was advanced under bronchoscopic guidance into the distal trachea and positioned appropriately above the carina.

Correct placement was confirmed by end-tidal carbon dioxide monitoring, bilateral breath sounds, and symmetric chest rise. The 9.0 cuffed tracheostomy tube was left in place with the cuff deflated to maintain an additional route for airway access should the newly placed endotracheal tube become dislodged. The patient remained sedated and mechanically ventilated and was transported back to the intensive care unit in stable condition.

## Discussion

Airway management in critically ill patients with severe infection presents challenges that extend beyond routine anatomic difficulty. Severe infection and sepsis are associated with systemic inflammation, capillary leak, and mucosal edema, all of which can significantly distort upper airway anatomy and compromise visualization [5]. In our case, these effects were compounded by prior airway manipulation and failed intubation, resulting in extensive edema, friable tissue, and active bleeding. Together, these factors obscured normal anatomic landmarks and narrowed the margin for safe airway intervention.

Infection-related airway edema may persist despite clinical stabilization and can be difficult to predict using standard preoperative airway assessments. Traditional predictors such as Mallampati classification and patient cooperation are often unavailable or unreliable in critically ill and sedated patients [6]. As demonstrated in this case, airway planning relied instead on the patient’s clinical history, physiologic reserve, and prior airway events. Recognizing these factors was essential in anticipating a high-risk airway and in shaping a cautious, anesthesia-driven strategy. 

A central principle highlighted by this case is the importance of airway preservation. In patients with limited cardiopulmonary reserve, particularly those with septic shock, loss of a functioning airway can rapidly result in severe hypoxia [7]. Rather than prioritizing rapid airway exchange, the anesthetic plan emphasized maintenance of continuous ventilation while escalating visualization in a very controlled manner. This approach allowed time for assessment, minimized additional airway trauma, and reduced the risk of complete airway loss.

The sequential use of airway devices in our case illustrates the value of flexibility over rigid adherence to a single technique. Initial video laryngoscopy provided insufficient visualization due to diffuse edema. Transitioning to an alternative video laryngoscope improved identification of the distal airway structures and enabled progression to a more precise modality. Importantly, fiberoptic bronchoscopy was used not as a blind rescue technique but as a targeted tool for confirmation and controlled tube placement once partial visualization was achieved. This sequencing maximized safety while minimizing unnecessary manipulation of an already inflamed airway. A strategy using direct laryngoscopy for routine airways and videolaryngoscopy for anticipated difficult airways was associated with a low rate of unanticipated failed intubation, with both videolaryngoscopy and fiberoptic bronchoscopy facilitating airway rescue when needed [8]. 

Fiberoptic bronchoscopy can also be valuable in distorted airways when used thoughtfully and in combination with other visualization tools [9]. In our case, it allowed direct confirmation of airway entry and precise placement of the endotracheal tube above the carina and even provided immediate physiologic confirmation through end-tidal carbon dioxide monitoring, bilateral breath sounds, and symmetric chest rise. This supports the role of fiberoptic techniques as tools for confirmation and refinement rather than sole rescue devices in unstable or poorly visualized airways [9]. In the setting of distorted airway anatomy caused by soft tissue swelling, fiberoptic bronchoscopy has been recommended as a primary technique to maintain airway security while preserving ventilation. This approach minimizes the risk of airway collapse in patients with limited airway reserve [9].

This case also highlights the anesthetic challenges of sepsis beyond its effects on hemodynamic stability. Patients with sepsis often have reduced tolerance for hypoxia, limited physiologic reserve, and increased risk with repeated airway attempts [7]. These factors support a strategy that prioritizes oxygenation, visualization, flexibility, and controlled progression over speed or technical simplicity. In these situations, anesthetic management requires a careful balance between securing a definitive airway and avoiding repeated or aggressive instrumentation, particularly when visualization is limited.

Although this case report describes a single patient, the principles illustrated are broadly applicable to similar high-risk scenarios. Patients with severe infection, airway inflammation, edema, and prior airway trauma require individualized airway plans that prioritize preservation of ventilation and avoidance of further airway injury. Rather than relying on a single best device or technique, success in these situations depends on an anesthesiologist's judgment, adaptability, and understanding of how systemic illness, such as sepsis, alters airway anatomy and physiology.

## Conclusions

Sepsis-related airway inflammation and prior airway trauma can significantly distort airway anatomy and increase the risk of airway loss during definitive management. In such settings, anesthetic success may depend less on any single device and more on thoughtful planning, preservation of an existing airway, and staged escalation guided by visualization. This case highlights how adapting airway strategy to the physiologic and inflammatory context of sepsis, with controlled progression and avoidance of rapid or blind intervention, may help mitigate risk in selected high-risk airway scenarios. 
